# Ethanol extracts of *Allium* sp. regulate cyclooxygenase-2 and E-cadherin expression in gastric cancer MKN74 cell line and enhance doxorubicin toxicity

**DOI:** 10.29219/fnr.v63.3449

**Published:** 2019-06-25

**Authors:** Agnieszka Korga, Marta Ostrowska, Magdalena Iwan, Małgorzata Skierucha, Aleksandra Józefczyk, Piotr Pawłowski, Jarosław Dudka, Ryszard Maciejewski, Robert Sitarz

**Affiliations:** 1 Department of Medical Biology, Medical University of Lublin, Lublin, Poland; 2 Department of Toxicology, Medical University of Lublin, Lublin, Poland; 3 Department of Anatomy, Medical University of Lublin, Lublin, Poland; 4 Surgical Oncology Department, Medical University of Lublin, Lublin, Poland; 5 Department of Pharmacognosy with Medicinal Plant Unit, Medical University of Lublin, Lublin, Poland

**Keywords:** Garlic, *Allium* sp, gastric cancer, cyclooxygenase 2, E-cadherin

## Abstract

**Background:**

Gastric cancer (GC) remains one of the leading causes of cancer-related death. Its aetiology is multifactorial, but the major risk factor is a high in salt diet. During gastric carcinogenesis, cadherin-1 (CDH1) down-expression and cyclooxygenase 2 (COX2) overexpression may be observed. The intensity of these alterations contributes to the GC invasion, its metastases and poor prognosis. As the diet plays a significant role in the aetiology of GC, it is reasonable to include the nutritional chemoprevention agents. One of the plant genus demonstrating chemoprotective properties is *Allium* genus, which includes garlic. The relationship between CDH1 and COX2 in GC cells treated with *Allium* species extract has never been evaluated.

**Methods:**

In this study, the MKN28 and MKN74 GC cell lines were treated with ethanol extracts of *Allium angulosum* L., *Allium lusitanicum* Lam., *Allium sativum* L. (from Malaysia and Poland), *Allium tibeticum* Rendle and *Allium ursinum* L. The cytotoxicity of the extracts and their influence on COX2 and CDH1 mRNA and protein expression were evaluated as well as their influence on doxorubicin’s (DOX) efficacy – a drug that has been used in GC treatment.

**Results:**

Among the tested species, ethanol extracts of *A. sativum* L. (Poland and Malaysia), *A. tibeticum* Rendle and *A. ursinum* L. influenced the levels of CDH1 and COX2, but only in the MKN74 cell line. Thus, it is possible that tumours with increased COX2 expression will be more susceptible to garlic treatment. Observed phenomenon was independent of *Allium* extract’s toxicity. In comparison to DOX, tested extracts were more toxic. Moreover, *A. sativum* revealed synergistic effect with the drug.

**Conclusion:**

In conclusion, the results indicate the potential application of *Allium* genus to GC chemoprevention and treatment support through CDH restoration and COX2 downregulation. This issue needs further investigations as it might be used in clinics.

## Popular scientific summary

- The gastric cancer (GC) incidence remains the third most common cause of cancer-related death. During gastric carcinogenesis cadherin-1 (CDH1) down-expression and cyclooxygenase 2 (COX2) overexpression may be observed what contributes to the GC invasion, its metastases and poor prognosis.
- We aimed to investigate for the first time the chemo preventive effect of ethanol extracts from various Allium species as well as the effect on COX2 and CDH1 expression relationship in the human GC cell lines, and doxorubicin toxicity.
- Our results indicate the potential application of Allium genus to chemo prevention and treatment support through CDH1 restoration and COX2 down regulation. However, the issue needs further in vivo investigations as in the future it might be used in clinics, possibly as a supplement to the chemotherapy.
- What is more, the tested extracts revealed cytotoxicity properties against GC cell lines and they had beneficial effect on DOX treatment.

Gastric cancer (GC) remains the third most common cause of cancer-related death worldwide (1). Despite advances in diagnosis and treatment, the 5-year survival rate is only around 20% (2, 3). Its aetiology is multifactorial, but the major risk factor is high content of nitrates and high salt intake. *Helicobacter pylori* infection also plays an important role. GC development involves multiple genetic and epigenetic alterations (2, 3). Despite advances in diagnosis, the disease is usually detected after invasion because of nonspecific symptoms in its early stages. Doxorubicin (DOX), an anthracycline chemotherapy agent, had been used as the gold standard for advanced GC since 1980 (4). However, DOX-based treatment is not currently recommended because of the frequent development of resistance and poor drug efficacy. As the diet plays a significant role in the aetiology of GC, it is reasonable to include nutritional chemoprevention agents in the diet.

Previous studies have shown properties of phytochemical compounds beyond their antioxidant activity. It turns out that natural compounds can also affect the proliferation, growth or metastasis of tumours thus being natural anticancer drugs. The amount of phytochemical compounds of daily diet may be the basis for a valuable cancer prevention strategy. One of the genus demonstrating chemoprotective properties is *Allium* genus. It comprises more than 600 species and belongs to the *Amaryllidaceae* family. They are plants of the northern hemisphere. For most species, the place of origin is Asia as well as Africa and North America (5). The best known and used, since ancient times, plant of this species is its characteristic representative “garlic” (*Allium sativum* L.). Wild (Bear) garlic (*A. ursinum* L.) reveals similar taste and healing properties. Mouse garlic (*Allium angulosum* L.) is important for its use as a spice, a vegetable and an ornamental plant in India (6). This area also includes *Allium tibeticum* Rendle which is the synonym of *A. sikkimense* Baker. *Allium lusitanicum* Lam. is a species that grows in southern Europe and Asian area. In the last three decades, studies focusing on garlic and its compounds have provided much evidence for their ability to prevent or treat various diseases. The genus shows antibacterial, antifungal, immunostimulating, antioxidant as well as chemoprotective properties and the ability to inhibit the growth of some cancer cells (7, 8). However, molecular mediators of these activities remain an intense area of research (9). *In vitro* and *in vivo* studies have shown that garlic demonstrates chemopreventive activity against cancer by inhibiting cell proliferation, arresting the cell cycle, inducing cellular apoptosis and blocking invasion and metastasis (10, 11).

Cyclooxygenase 2 (COX2) is an isoform of COX enzymes that play an important role in the development and progression of GC (12–14). COX enzymes are induced in response to pro-inflammatory cytokines and growth factors. They are responsible for the catalysis of prostanoid biosynthesis, thus playing an important role in regulating various cellular functions under physiological and pathological conditions, including the process of tumour formation (15–17). Studies have shown that COX2 is involved in tumour invasion and metastasis, among others, by reducing the expression of cadherin E (CDH). CDH is a cell adhesion molecule whose expression decreases during tumour progression. The most important function of CDH is inhibition of tumour invasion and metastasis. Thanks to this, the normal epithelial tissue architecture is maintained. An abnormal expression and function of CDH has been observed in several epithelial cancers, including GC (18, 19). The inverse relationship between CDH1 and COX2 and its molecular mechanism in tumour cells was demonstrated for the first time in non-small cell lung cancer, in which the inhibition of tumour COX2 with celecoxib led to increased expression of CDH1 (20). A similar effect was also found in colon, bladder as well as head and neck cancer cells (21–23). For the first time, our research team demonstrated this relationship in GC (24). COX2 over-expression has been found in many types of tumours, including GC (12, 25), what contributes to its invasion, metastasis and poor prognosis (26, 27). Studies that showed COX2 upregulation in GC confirm the fact that it is reasonable to investigate the chemopreventive role of COX2 inhibitors in this cancer. Many animal and epidemiological studies have provided evidence that non-steroidal anti-inflammatory drugs and selective COX2 inhibitors suppress tumourgenesis (27–30). Previous research articles indicate that COX2 inhibitors not only exhibit chemoprophylactic effect in GC but also have chemotherapeutic potential (31). There are also many reports on the effect of COX2 inhibitors on the efficacy of anticancer drugs, but the results are not consistent (32).

*Allium* genus plants have been known for their anti-inflammatory properties for centuries (33). It has been proven that garlic may inhibit COX2 activity (34). For this reason, the study aimed to investigate for the first time the chemopreventive effect of ethanol extracts from various *Allium* species as well as their effect on COX2 and CDH1 expression in the human GC cell lines, and DOX toxicity.

## Materials and methods

### Plant material

Selected species of fresh *Allium* (bulbus and radix) were obtained from the Botanical Garden Chair and Department of Pharmacognosy with Medicinal Plant Unit, Faculty of Pharmacy, Medical University of Lublin (Poland) and included *A. angulosum* L. *A. lusitanicum* Lam., *A. sativum* L. (from Poland, PL), *A. tibeticum* Rendle and *A. ursinum* L. The seed came from an exchange between botanical gardens. *A. angulosum* L. – The Tropical Greenhaus in Ljubljana, Slovenia, *A. lusitanicum* Lam. – Botanischer Garten der Universiteat Graz, Austria, *A. tibeticum* Rendle I Grădina Botanica Jibou, Vasile Fati, Romania, *A. ursinum* L., *A. sativum* L. – Medicinal Herbs Centre, Facultas Medica – Universites Masarykiena, Brno, Czech Republic. In the study, we also used fresh (2-day) tubers and roots of *Allium sativum* L. purchased from a market in Singapore (Malaysia, MY). The plant material was identified by specialists from the Department. Fresh substances have been chopped to a medium crushed content and extracted with 65% ethanol. The extraction was carried out by 24-h maceration with automatic shaking (250 rpm/min) for the last hour. After extraction, the extracts were soaked and supplemented with extractors to a volume in which the ratio of the mass of plant substance (g) used to the sample volume (mL) was 1:5. Extracts were used as stock solutions.

### Cell culturing and treatment

Human GC cell lines MKN28 and MKN74 were acquired from the Department of Pathology at the University Medical Center in Utrecht, Holland. The cells were maintained in RPMI medium (Corning, USA) supplemented with 10% foetal bovine serum (PAA Laboratories, Canada) in a humidified atmosphere of 5% CO_2_ at 37°C. Prior to the experiments, all cell lines have been tested for the presence of mycoplasma using LookOut^®^ Mycoplasma PCR Detection Kit (Sigma Aldrich, USA). Tested extracts were added to the cell cultures in final concentration 0.3 µg/mL (ethanol concentration did not exceed 0.1% in the culture medium) and incubated for 24 and 48 h.

In the second part of the study where the influence of extracts on DOX toxicity was evaluated, the MKN74 cells were incubated with 1 μM DOX (EBEWE Pharma, Austria) and tested with extracts alone or combined (DOX + single extract). The concentration of DOX was based on reported clinically achievable plasma concentrations (35). Extract was selected on the basis of previous gene expression analysis (unpublished data) – we chose *A. sativum* MY and PL, *A. ursinum* and *A. tibeticum* that upregulated *CHD1* and downregulated *COX2* expression at the same time.

### The cytotoxicity analysis

The cytotoxicity of the tested extracts was evaluated with the MTT (3-(4,5-dimethylthiazol-2-yl)-2,5-diphenyltetrazolium bromide) test, using the MTT Cell Proliferation Assay Kit (Thermofisher, USA). The test principle is based on live cells ability to reduce orange tetrazolium salt to water-insoluble purple formazan crystals. Cells were inoculated into 96-well plate in the concentration of 2.5 × 10^5^ cells/mL. Tested compounds were added when 70–80% confluence was achieved. MTT solution (4 mg/mL) was added to the culture after 24 and 48 h of incubation with extracts. After following 4-h incubation, the medium with MTT salt was removed and then the formed crystals were dissolved in dimethyl sulfoxide. The solution absorbance was measured at 540 nm, using PowerWave™ microplate spectrophotometer (Bio-Tek Instruments, USA). Each experiment was conducted three times and was measured in triplicates.

### mRNA expression

Cells were inoculated into a 6-well plate in the concentration of 2.5 × 10^5^ cells/mL. Tested compounds were added when 70–80% confluence was achieved. After 24 and 48 h, incubation cells were harvested using trypsin. RNA was isolated from the cells using Syngen Blood/Cell RNA Mini Kit (Syngen Biotech, Poland) according to the manufacturer’s protocol. cDNA was synthesised using High Capacity cDNA Reverse Transcription Kit (Thermofisher, USA) following the manufacturer’s instructions. Quantitative real-time PCR was performed in triplicate using TaqMan^®^ Gene Expression Assays: Hs01023894_m1 (*CDH1*), Hs00153133_m1 (*COX2*) and Hs02758991_g1 (*GAPDH*) (Thermofisher, USA) with 7500 Fast Real-Time PCR system (Applied Biosystem, USA). Relative gene expression levels were determined using ΔΔCt method and presented as the mean fold change (RQ = 2^− ΔΔCt^). GAPDH was used as a reference gene.

### Protein expression

Cells were inoculated into a 6-well plate in concentration of 2.5 × 10^5^ cells/mL. Tested compounds were added when 70–80% confluence was achieved. After 24- and 48-h incubation, the cells were washed with PBS and then were incubated in lysis buffer (150 mM NaCl, 1% Triton X-100, 0.1%SDS, 50 mM Tris pH = 8) for 30 min in 4°C. The lysates were centrifuged (20 min, 12,000 rpm), and the protein concentration in the supernatants was determined by the Bradford method. The extracted protein (20 μg) was loaded in a polyacrylamide gel (NuPAGE Bis-Tris Gels, Invitrogen, USA) and 50-min electrophoretic separation was performed under reducing conditions with XCellSureLock instrument (Invitrogen, USA) at a constant voltage of 200 V. After separation, the gel and the nitrocellulose membrane (Invitrogen, USA) were placed between two layers of the filter paper and put between two electrodes of the XCell II Blot Module electrotransfer apparatus (Invitrogen, USA). The transfer was conducted for 60 min at a constant voltage of 30 V. Blots were developed using Western Breeze Chromogenic Detection Kit (Invitrogen, USA). Nonspecific antibody-binding sites were blocked and the membrane was incubated in the solution of the primary antibody: CDH1 antibody (H-108), COX2 antibody (H-62) and β-actin antibody (N-21) (Santa Cruz Biotechnology, US). After washing, the membrane was incubated with the secondary antibody combined with alkaline phosphatase. The last step was to mark the sites of reaction with antigen by the reaction of alkaline phosphatase and chromogen. The relative intensities of bands were quantified using the 1D Image Analysis Software program (Kodak, USA), and all the values were normalised to the intensities of the respective β-actin signal that was used as a loading control (Abcam, USA).

### Statistical analysis

The results were analysed statistically in the STATISTICA vs. 13 application (StaftSoft, Poland). Data were calculated as mean ± SD. To compare two groups, Student’s *t*-test was used. To compare more than two groups, the one-way analysis of variance (ANOVA) and *post hoc* multiple comparisons on a basis of Tukey’s HSD test were used. All parameters were considered statistically significantly different if *P* values were less than 0.05.

## Results

### Toxicity of plant extracts

Cytotoxicity of the tested extracts was different for MKN28 and MKN74 cell lines. *Allium angulosum* and *A. ursinum* were toxic for MKN28 cells (48.60 ± 4.46 and 66.77 ± 3.00% viability after 48 h incubation respectively, see Table 1). All tested extracts revealed toxicity towards MKN74 cells; however, it was differentiated – the highest toxicity was observed after 48 h treatment with *A. ursinum* extract (31.55 ± 2.04% viability), and the lowest was observed after 48 h treatment with *A. tibeticum* (84.56 ± 6.60% viability) (Table 1).

**Table 1:** Treated MKN28 and MKN74 cells’ viability (% of control) after 24 and 48 h

Garlic extract (0.3 μg/mL)	MKN28						MKN74					
	24 h			48 h			24 h			48 h		
*A. angulosum*	75.194	±	7.482*	48.598	±	4.465***	40.147	±	2.626 ***	34.850	±	2.797***
*A. sativum* PL	110.888	±	16.681	93.474	±	3.460	59.941	±	4.602***	64.969	±	2.604***
*A. sativum* MY	101.550	±	4.234	100.431	±	4.817	74.366	±	0.721***	71.102	±	1.877***
*A. ursinum*	61.135	±	1.548***	66.775	±	3.003***	35.339	±	4.478***	31.546	±	2.037***
*A. tibeticum*	87.174	±	1.898	96.872	±	10.001	74.336	±	3.111***	84.582	±	6.601**
*A. lusitanicum*	86.786	±	6.116	84.520	±	2.049*	86.903	±	4.752**	67.346	±	7.153***

Abbreviations: A. – *Allium*; PL – Poland; MY – Malaysia; SD – standard deviation.

*** *P* < 0.001

** *P* < 0.01

* *P* < 0.05 vs. relevant control (mean ± SD; MKN28 control 24 h: 100.000 ± 3.763; 48 h: 99.946 ± 2.73; MKN74 control 24 h: 100.029 ± 6.731; 48 h: 100.023 ± 7.941)

### E-cadherin and cyclooxygenase 2 expression

**Fig 1:**
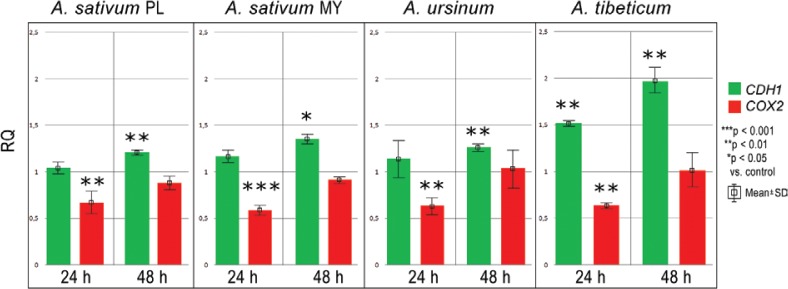
Chart bar illustrating the results of *CDH1* and *COX2* mRNA expression in MKN74 cell line treated with *Allium sativum* PL (Poland), *Allium sativum* MY (Malaysia), *Allium ursinum* and *Allium tibeticum* after 24 and 48 h. Data are presented as the mean RQ ± SD. The statistical significance of differences between control and treated cultured was presented as *P ≤ 0.05, **P ≤ 0.01, ***P ≤ 0.001.

**Fig 2:**
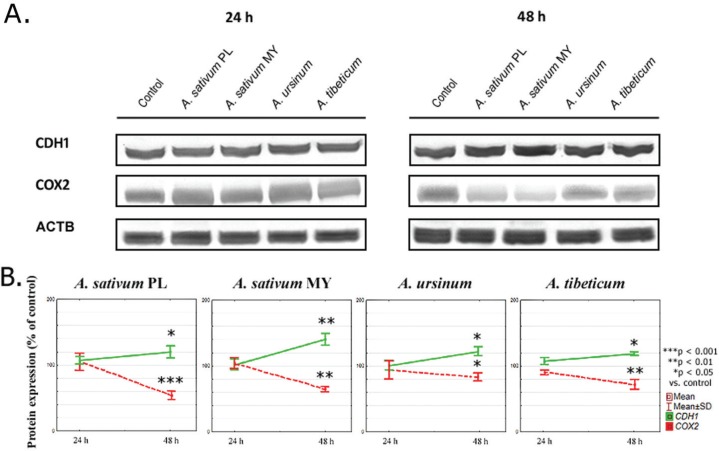
Graphs illustrating the results of: (A) Representative western blots from whole cell extract showing CDH1 and COX2 protein levels, and (B) CDH1 and COX2 protein expression. Data are presented as the mean percentage of control ±SD. The statistical significance of differences between control and treated cultured was presented as *P ≤ 0.05, **P ≤ 0.01, ***P ≤ 0.001.

In our study, four ethanol extracts of three *Allium* species (*A. sativum* PL and MY, *A. ursinum* and *A. tibeticum*) influenced both CDH1 and COX2 levels in MKN74 human gastric cell line (Figs. 1 and 2) in contrast to MKN28 cell line, where this correlation was not observed.

### mRNA expression

After 24 h of incubation, *CDH1* mRNA expression was elevated after treating with *A. sativum* MY and *A. tibeticum* extracts (RQ = 1.17 ± 12 and 1.52 ± 0.03, respectively) in comparison to the control cells. After 48 h, the expression was even higher for *A. sativum* PL, *A. sativum* MY, *A. tibeticum* and *A. ursinum* extracts (see Fig. 1).

*COX2* mRNA expression was considerably decreased at 24 h. At 48 h, it increased and did not exceed the control level for *A. sativum* PL ethanol extract (RQ = 0.88 ± 0.06) and *A. sativum* MY ethanol extract (RQ = 0.91 ± 0.05). In general, ethanol extracts influenced *COX2* mRNA expression at 24 h, and the intensity of this effect decreased over time (Fig. 1).

### Protein expression

To evaluate whether changes in mRNA expression were translated into protein expression, we conducted western blotting analysis. After 24 h of incubation, the proteins levels of both CDH1 and COX2 changed significantly after *A. tibeticum* extract treatment (107.48 ± 3.26 and 90.32 ± 3.67% respectively, Fig. 2). After 48 h, increased CDH1 expression and decreased COX2 expression were observed.

As one can compare, *COX2* mRNA expression was decreased after 24 h in comparison to the control, then increased in time. On the contrary, the COX2 protein expression significantly lowered after 48 h when compared to the control level.

### Influence of plant extracts on DOX toxicity

**Fig. 3:**
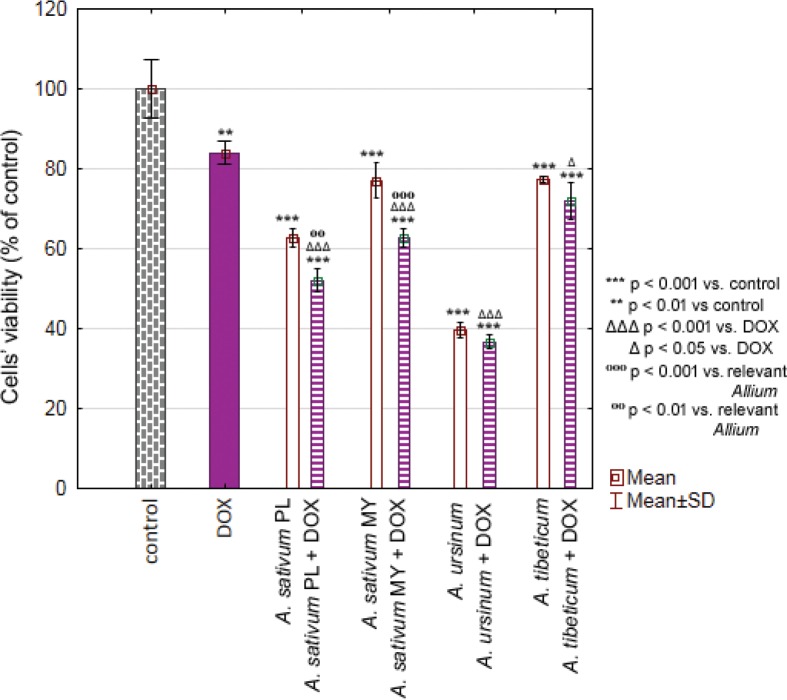
Chart bar illustrating the results of MTT assay carried out with MKN74 cell line treated with doxorubicin (DOX), *Allium sativum* PL (Poland), *Allium sativum* MY (Malaysia), *Allium ursinum* and *Allium tibeticum* as well as DOX + single extract after 24 h. Data are presented as the mean ± SD. Results were compared with control, DOX or relevant *Allium* extract.

The week toxicity of DOX towards MKN74 cell line has been revealed (84.03 ± 2.88%). Selected extracts were even more toxic than DOX alone (*A. sativum* PL: 621.76 ± 2.34%; *A. sativum* MY: 77.10 ± 4.39%; *A. ursinum*: 39.57 ± 1.91%; *A. tibeticum*: 77.30 ± 0.83%) and their simultaneous treatment did not diminish observed toxicity. Moreover, synergistic effect of DOX and *A. sativum* (PL: 52.12 ± 2.83% and MY: 62.63 ± 2.31%) was observed (Fig. 3).

## Discussion

Epidemiologically, GC is one of the most common malignancies in humans. It is the sixth malignant tumour in incidence and the third in terms of mortality (1). Numerous studies have shown that vegetables from the *Allium* family, mainly garlic, may play an important role in the prevention of cancer (36, 37) and its treatment (38). One of the possible mechanisms of this phenomenon is the CDH expression restoration and thus inhibition of tumour invasion and metastasis (39) – CDH is an essential protein in the cell–cell adhesion and its downregulation and mutations favour cancer development and metastasis (40). This process has been previously reported in prostate and oral squamous cancer cells (41, 42). However, to the best of our knowledge, our study is the first concerning garlic-induced CDH restoration as a potential application in GC treatment.

The second molecule that plays an important role in the development and progression of GC is COX2. Its expression has been demonstrated to be elevated in this kind of tumour. For this reason, its inhibition has become one of the strategies of GC prevention and treatment. For the first time, our research team showed an inverse relationship between CDH and COX2 in GC (24). Garlic and other *Allium* species representatives are known anti-inflammatory agents and act as COX2 inhibitors. To our knowledge, the influence of garlic on COX2 expression as well as the relationship between CDH and COX2 after treatment with *Allium* extracts in GC has never been investigated. In our study, the CDH1 and COX2 expression depended on the time of exposure to garlic extracts. In all tested extracts, a positive effect of increased CDH1 expression intensified in 48 h. Largest drop in COX2 expression was observed in 24 h. These findings suggest that the effect of garlic extracts on GC cells is reversible. It is worth noting that the durability of this effect may depend on the tumour cell line – in Moden et al. (43) study, breast cancer cells supplemented with garlic extracts remained in growth arrest for 3 weeks.

The question is why the influence of the examined extracts was observed only in MKN74 cells. A possible explanation can be the difference in the COX2 expression level in both cell lines. As Saukkonen et al. (44) revealed, MKN74 cell line is characterised by higher COX2 expression than MKN28, both at the mRNA and protein levels, and thus is more sensitive. Therefore, it is possible that tumours with increased COX2 expression will be more susceptible to garlic treatment.

The pattern of gene expression is not always related to cytotoxicity. However, studies indicate that COX2 inhibitors are associated not only with chemoprophylactic effects but also with the chemotherapeutic potential in GC. Both COX-dependent and COX-independent pathways play a major role in the anti-tumour efficacy of COX2 inhibitors (31). For this reason, we evaluated the toxicity of all obtained extracts against the MKN28 and MKN74 cell lines and compared them with the observed inverse relationship between CDH and COX2. MKN28 cells were sensitive to *A. angulosum* and *A. ursinum* ethanolic extracts (Table 1). All tested extracts were toxic to MKN74 cells, of which *A. angulosum* and *A. ursinum* showed the strongest effect (Table 1). These results suggest that cytotoxicity was independent of changes in the CDH1 and COX2 expression patterns. There have been reports on the anti-tumour activity of *A. sativum* and *A. ursinum* (45–48); however, for the first time we revealed the cytotoxic activity of *A. angulosum* on GC.

DOX is an anthracycline chemotherapy agent that has been used as the gold standard for advanced GC since 1980 (4). However, DOX-based treatment is not recommended in chemotherapy for GC because of the frequent development of resistance and poor drug efficacy. The study assessed the effect of the selected extract on the toxicity of DOX relative to the MKN74 cell line. It is believed that DOX exerts anti-tumour activity through two different pathways – intercalation within DNA base pairs causing DNA strands breakage as well as the production of reactive oxygen species (ROS) that cause death in both cancer and normal cells. Oxidative stress is more often considered in the context of anthracycline cardiotoxicity (31, 49), and many studies have shown cardioprotective activity of plants from *Allium* species, especially garlic (50). However, their effect on DOX anti-cancer activity has not been studied. *Allium* species belong to natural antioxidants, and the effect of this group of compounds on the anticancer drug, including DOX, remains controversial (51). Because of the DOX oxidation mechanism, we could suppose weakening the antitumor activity of the drug. On the other hand, synergistic effects of DOX on antioxidants have been noticed. Our study showed that the ethanol extracts of *A. tibeticum* and *A. ursinum* were more toxic than DOX alone and the concomitant treatment did not affect its toxicity. In addition, *A. sativum* (PL and MY) showed a synergistic effect with the clinical dose of DOX.

This suggests that ROS production is not critical to DOX anti-tumour activity in this situation. This is consistent with previous observations that ROS-induced apoptosis of tumour cells occurs only in supraclinical doses of anthracyclines (52).

## Conclusions

Our results indicate the potential application of *Allium* genus to chemoprevention and treatment support through CDH1 restoration and COX2 downregulation. However, the issue needs further *in vivo* investigations as in the future it might be used in clinics, possibly as a supplement to the chemotherapy. Moreover, the tested extracts revealed cytotoxic properties against GC cell lines and they had beneficial effect on DOX treatment.

## Data Availability

The data sets used and/or analysed during this study are available from the corresponding author on reasonable request.
